# Influence of Oxide Glass Modifiers on the Structural and Spectroscopic Properties of Phosphate Glasses for Visible and Near-Infrared Photonic Applications

**DOI:** 10.3390/ma13214746

**Published:** 2020-10-23

**Authors:** Marta Kuwik, Joanna Pisarska, Wojciech A. Pisarski

**Affiliations:** Institute of Chemistry, University of Silesia, Szkolna 9, 40007 Katowice, Poland; wojciech.pisarski@us.edu.pl

**Keywords:** phosphate glass, structure, oxide modifier, visible luminescence, 1.5 μm emission

## Abstract

The effect of oxide modifiers on multiple properties (structural and spectroscopic) of phosphate glasses with molar composition 60P_2_O_5_-(10−x)Ga_2_O_3_-30MO-xEu_2_O_3_ and 60P_2_O_5_-(10−y)Ga_2_O_3_-30MO-yEr_2_O_3_ (where M = Ca, Sr, Ba; x = 0, 0.5; y = 0, 1) were systematically examined and discussed. The local structure of systems was evidenced by the infrared (IR-ATR) and Raman spectroscopic techniques. The spectroscopic behaviors of the studied glass systems were determined based on analysis of recorded spectra (excitation and emission) as well as luminescence decay curves. Intense red and near-infrared emissions (1.5 μm) were observed for samples doped with Eu^3+^ and Er^3+^ ions, respectively. It was found that the value of fluorescence intensity ratio R/O related to ^5^D_0_→^7^F_2_ (red) and ^5^D_0_→^7^F_1_ (orange) transition of Eu^3+^ ions depends on the oxide modifiers MO in the glass host. However, no clear influence of glass modifiers on the luminescence linewidth (FWHM) was observed for phosphate systems doped with Er^3+^ ions. Moreover, the ^5^D_0_ and ^4^I_13/2_ luminescence lifetimes of Eu^3+^ and Er^3+^ ions increase with the increasing ionic radius of M^2+^ (M = Ca, Sr, Ba) in the host matrix. The obtained results suggest the applicability of the phosphate glasses with oxide modifiers as potential red and near-infrared photoluminescent materials in photonic devices.

## 1. Introduction

Over the past years, inorganic glass systems have been paid attention due to their interesting properties and possible applications as fibers and optical broadband amplifiers, lasers, optical temperature sensors and generators of white light [1,2,3,4,5]. The composition of the glass host matrices influences significantly the structural, electrical, optical and physical properties of glass systems [6,7,8,9,10].

Particularly noteworthy are the glass modifiers such as alkaline earth metal oxides (MO = MgO, CaO, SrO, BaO) that strongly influence the properties of glass systems [11,12,13,14]. Previously published results indicate that the depolymerization of the glass network due to the incorporation of alkaline earth oxides into the glass matrix promotes the formation of non-bridging oxygen (NBO) groups [15]. Moreover, Pavić et al. [16] stated that modifiers like MgO, CaO as well as BaO influence some properties (electrical, dielectric and spectroscopic) of Fe_2_O_3_ doped into phosphate glasses. The effect of oxide modifiers on the luminescence properties and decay measurements of Ln^3+^ ion-doped inorganic glass matrices has been the subject of numerous studies. Ratnakaram et al. [17] reported the role of glass modifiers in the luminescence spectra and kinetics of Eu^3+^-doped lithium fluoroborate systems. The optical properties of Er^3+^-doped alkaline-earth borotellurite glasses were studied by Swapna et al. [18]. Also, the effect of MgO, CaO, SrO and BaO on white luminescence of borate systems doped with Dy^3+^ ions has been proven [19]. According to the results obtained for glasses, the addition of modifier oxides into the glass host can provide important advantages. For instance, the lithium antimonate and antimony borate glasses with calcium oxide exhibit better lasing action in comparison to samples with other studied glass modifiers. This is related to the larger value of the branching ratio (β) for CaO modifier glasses [20]. Moreover, comparatively low non-radiative losses for CaO mixed systems are found to be the reason for the high luminescence efficiency of these glasses [21]. Similar results were confirmed for P_2_O_5_-Pb_3_O_4_-ZnO-CaO systems doped with dysprosium ions [22]. On the other hand, Nd^3+^-doped lithium fluoroborate glass with MgO shows a higher stimulated emission cross-section than systems with CaO, PbO or CdO [23].

Among the different inorganic glass systems, phosphate glasses have been studied for numerous industrial applications [24,25] and for broad optical applications in the range of visible luminescence [26,27,28,29,30] and infrared luminescence [31,32,33,34,35]. These glass materials containing rare earth ions such as Nd^3+^, Yb^3+^, Er^3+^ can be successfully used for high-power laser systems, optical amplifiers and optical sensors due to their excellent luminescent properties [36,37,38,39,40,41,42]. Special attention has been paid to phosphate glasses containing erbium ions [43,44,45,46]. The thermal, structural and luminescence properties of Er^3+^-doped phosphate glasses modified by Al_2_O_3_, TiO_2_ and ZnO have been examined [47]. Ga_2_O_3_ also belongs to the components playing an important role as an intermediate metal oxide, with is able to serve both as a glass-former and a glass-modifier in inorganic glasses [48]. In general, Ga_2_O_3_-based glasses possess quite good IR transparency and can be attractive glass-hosts for wideband transparent windows, achromatic lenses or luminescent materials emitting visible light or near-infrared radiation [49]. In particular, the glass thermal stability and some spectroscopic parameters of rare earth ions are improved significantly in the presence of Ga_2_O_3_. The experimental results for Er^3+^-doped borobismuth glasses clearly suggest that these thermal and spectroscopic parameters are optimal at Ga_2_O_3_ = 8 mol% [50], when gallium oxide plays the role as a glass-network-modifier.

It is well known that glasses based on P_2_O_5_ are attractive optical materials due to their exceptional characteristics like a high transparency in a wide spectral region, low melting point and low refractive index [51,52]. Furthermore, phosphate systems doped with higher concentrations of rare earths or transition metal ions are amorphous, in contrast to other oxide glasses [53]. The relatively large phonon energy and hygroscopic properties of P_2_O_5_ contribute to the lower intensity of luminescence for Ln^3+^ ions in phosphate glasses. For that reason, the applications of phosphate systems in the development of photonic devices may be limited [54]. However, the incorporation of modifier oxides into the phosphate glasses could improve the spectroscopic properties of systems based on P_2_O_5_. To the best of our knowledge, the structural and luminescence properties of P_2_O_5_-Ga_2_O_3_ glasses with alkaline earth metal oxides (MO) are not frequently examined. Therefore, it is interesting to thoroughly research the multiple properties (structural and spectroscopic) of phosphate glass systems with oxide modifiers as potential photoluminescent materials in photonic devices.

In this paper, we present results for phosphate glasses with oxide glass modifiers MO (where MO = CaO, SrO, BaO). The local structure of systems was evidenced by the infrared and Raman spectroscopic techniques. It was presented that the recorded bands due to the stretching modes of the P–O bonds depend on the type of oxide modifier used. The conversion of phosphate tetrahedral structural units Q^n^ and the depolymerization of the glass network occur when glass modifiers are changed. Moreover, the spectroscopic properties of optical active dopants (Eu^3+^ and Er^3+^) were determined based on analysis of registered luminescence spectra and luminescence decay curves. Their properties were compared from the point of view of the alkaline earth oxides in the glass host matrix. It was found that the intensity of the red and orange emissions of glasses doped with Eu^3+^ ions depends on the oxide modifier, whereas no clear influence of glass modifiers on the luminescence linewidth for 1.5 μm emission was observed for systems doped with Er^3+^ ions. The ^5^D_0_ and ^4^I_13/2_ luminescence lifetimes of Eu^3+^ and Er^3+^ ions increase with the increasing ionic radius of M^2+^ (M = Ca, Sr, Ba) in the glass host matrix. Based on that result, important for future applications, we can choose systems with good optical behaviors, which are potential red and near-infrared photoluminescent materials in photonic devices.

## 2. Materials and Methods

Phosphate glass systems singly doped with Eu^3+^ and Er^3+^ ions with the molar composition 60P_2_O_5_-(10−x)Ga_2_O_3_-30MO-xEu_2_O_3_ and 60P_2_O_5_-(10−y)Ga_2_O_3_-30MO-yEr_2_O_3_ (where M = Ca, Sr, Ba; x = 0, 0.5; y = 0, 1) were synthesized by conventional melt-quenching method using oxide components of high purity (99.99%, Aldrich Chemical Co., St. Louis, MO, USA). In the first step, the appropriate amounts of metal oxides were mixed/ground in an agate ball mill. Because of the hygroscopic properties of the glass network former P_2_O_5_, the glass samples were fabricated in a glow box under a protective atmosphere of dried argon. The samples were melted in a corundum crucible at 1100 °C for 30 min in an electrical furnace. In order to eliminate internal mechanical stresses, each sample was quenched and annealed below the glass transition temperature (Tg). Next, the obtained systems were slowly cooled to room temperature. Transparent glasses were shaped and polished to meet the requirements for optical measurements. The prepared samples were around 10 mm × 10 mm dimension and 2 mm in thickness.

IR spectroscopy and Raman spectroscopy were used to examine the local structure of studied glasses undoped with rare earth ions. The infrared spectra were performed using the ATR technique. Spectral measurements were performed on a Nicolet^TM^ iS^TM^ 50 FT-IR spectrometer (Thermo Scientific, Waltham, MA, USA) equipped with a diamond attenuated total reflectance (ATR) module. The infrared spectra were recorded in the spectral range of 1400–400 cm^−1^ with a resolution of 4 cm^−1^. The Raman spectra were obtained using a Thermo Scientific™ DXR™2xi Raman imaging microscope (Thermo Scientific, Waltham, MA, USA). The data were collected using a 455 nm laser and the power on samples was a 5 mW. The Raman spectra were registered with a resolution of 2 cm^−1^.

Optical measurements were carried out on a Photon Technology International (PTI) Quanta-Master 40 (QM40) UV/VIS Steady State Spectrofluorometer (Photon Technology International, Birmingham, NJ, USA) coupled with tunable pulsed optical parametric oscillator (OPO), pumped by a third harmonic of a Nd:YAG laser (Opotek Opolette 355 LD, OPOTEK, Carlsband, CA, USA). The laser system was equipped with a double 200 mm monochromator, xenon lamp as a light source, a multimode UVVIS PMT (R928) (PTI Model 914) detector and Hamamatsu H10330B-75 (Hammatasu, Bridgewater, NJ, USA) detector controlled by a computer. Excitation and emission spectra were registered with a resolution 0.5 nm. Luminescence decay curves were recorded and stored by a PTI ASOC-10 (USB-2500) oscilloscope. All structural and optical measurements were carried out at room temperature. 

## 3. Results and Discussion

### 3.1. Structural Properties of Phosphate Glasses

In agreement with the literature, phosphate glass matrices can be described by four different structures Q^n^. The first is a three dimensional cross-linked network of Q^3^ tetrahedra. The second is a polymer-like metaphosphate chain of Q^2^ structural units. There are also glasses based on Q^1^ and Q^0^ tetrahedra, which represent pyrophosphate and orthophosphate anions, respectively [55]. Infrared and Raman spectroscopy reveal important information regarding the modification in structural units Q^n^ with a change in the glass composition. According to Moustafa and El-Egili the stretching vibrations (asymmetric and symmetric) characteristic of phosphate lattices are active in the infrared as well as Raman spectra [56]. Most studies devoted to the Raman spectra of glass systems based on P_2_O_5_ suggest that intense bands are related to the symmetric stretches, whereas the infrared spectra intense bands correspond to asymmetric stretches.

**Figure 1:**
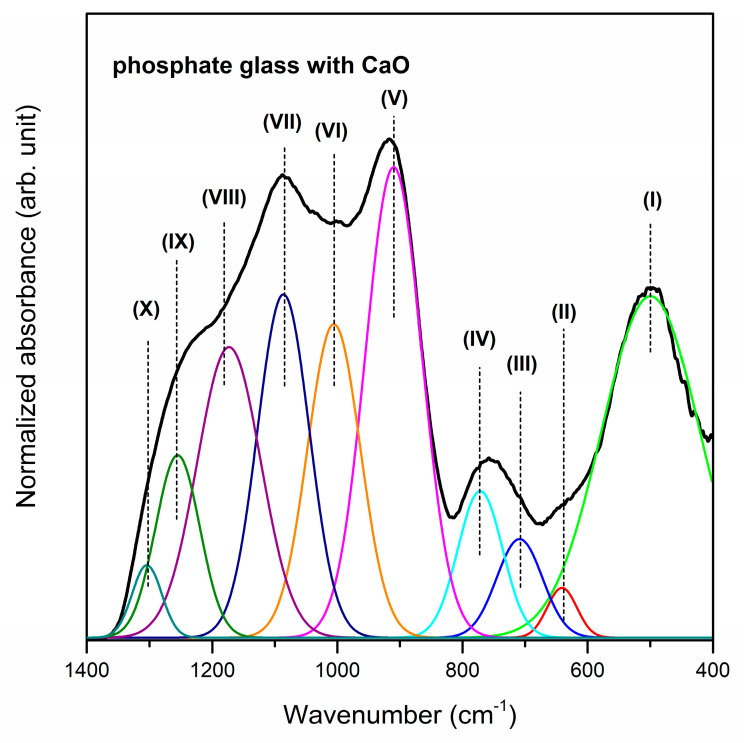
Deconvoluted near-infrared spectra of phosphate glass with CaO as oxide glass modifier.

**Figure 2:**
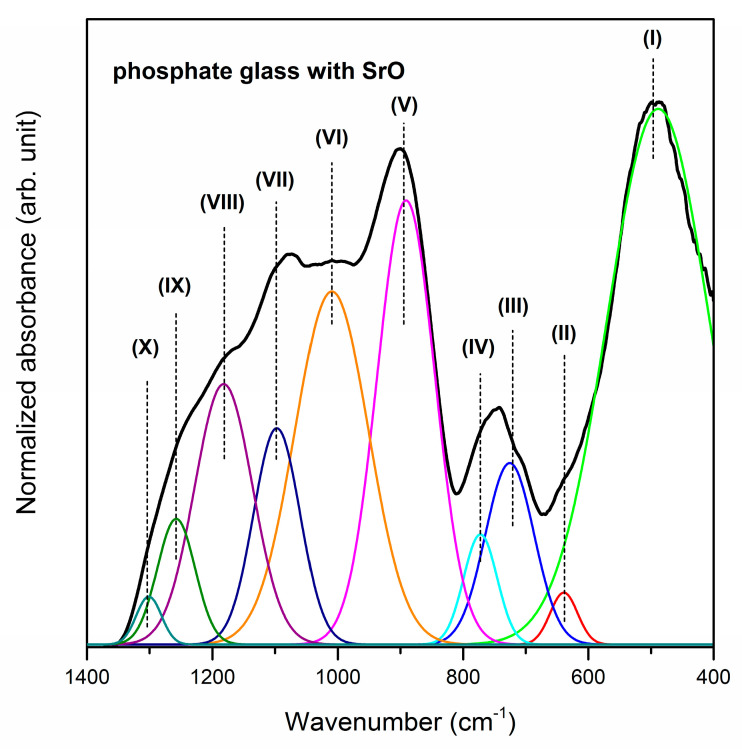
Deconvoluted near-infrared spectra of phosphate glass with SrO as oxide glass modifier.

**Figure 3:**
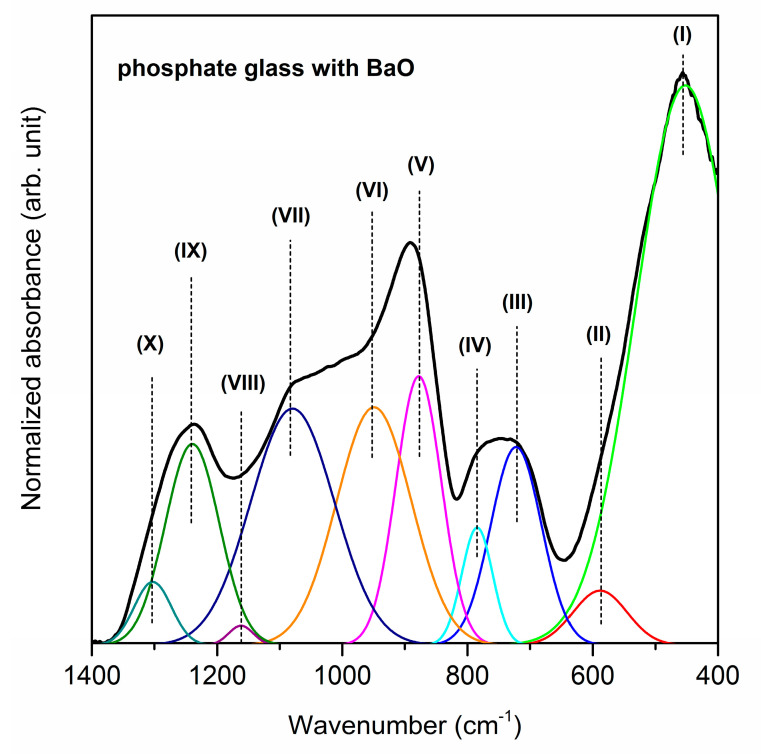
Deconvoluted near-infrared spectra of phosphate glass with BaO as oxide glass modifier.

Figure 1, Figure 2 and Figure 3 show the near-infrared spectra registered for phosphate systems with glass modifiers in 400–1400 cm^−1^ spectral region. In order to better understand the effect of oxide modifiers MO (calcium oxide, strontium oxide and barium oxide) addition on the phosphate glass structure, the deconvolution of the infrared spectra of all samples was carried out. As reported in the literature, the band (I) located at about 499/488/452 cm^−1^ is due to harmonics of bending vibrations of O=P–O linkages [57]. It is important to note that the areas of this band increase in comparison to other IR bands when oxide modifiers MO change in direction CaO < SrO < BaO. The next band centered at 640/639/588 cm^−1^ is attributed to the P–O–P bending vibration and stretching of P–O–P mode [58]. Moreover, the IR band (II) is characteristic of the stretching vibration of M–O–P bonds [59]. On the other hand, the previous results for PbO-Ga_2_O_3_-P_2_O_5_ glass [60] and Ga_2_O_3_-P_2_O_5_ glass systems [61] suggest that in this frequency region the IR band corresponding to vibrations localized on GaO_4_ tetrahedra can be observed at about 610 cm^−1^ and 640 cm^−1^, respectively. It was stated that the band located in the 708–722 cm^−1^ frequency region corresponds to the symmetric stretching of P–O–P linkages in between Q^1^ and Q^2^ units [58], whereas the infrared band near at ~780 cm^−1^ is due to the symmetric stretching mode of P–O–P bonds [62]. An increase in the intensity of the band (III) at the expense of band (IV) could be associated with the conversion of Q^2^ to Q^1^ units that is occurring with a change of oxide modifiers in the glass host. 

It is clearly seen from Figure 1, Figure 2 and Figure 3 that broad and unresolved infrared bands are located in the 800–1350 cm^−1^ frequency region. Furthermore, the analysis of this frequency region indicates the effect of calcium oxide, strontium oxide and barium oxide on the structure of phosphate systems. The bands due to asymmetric stretching vibrations of bridging oxygen atoms in P–O–P bonds [63] and asymmetric stretching vibrations of the P–O–P linkage of Q^1^ and Q^2^ tetrahedra with non-bridging oxygen are located at about 900 cm^−1^ [64]. The decrease in the relative area of the band for a sample with BaO as oxide modifier may be associated with the formation of the P–O–Ba bonds at the expense of the rest of the P–O–P linkages. Moreover, the P–O–P asymmetric stretching band (V) shifts to lower frequencies with a change of oxide modifier MO in direction CaO < SrO < BaO as a result of depolymerization of the glass network [65].

The bands in the 949–1009 cm^−1^ and 1079–1097 cm^−1^ frequency regions are related to asymmetric stretching vibrations of PO_4_^3−^ structural group and symmetric stretching vibrations of PO_4_^3−^ tetrahedral (PO^−^ ionic group) [66] or PO_3_^2−^ symmetric stretching vibrations in Q^1^ structure, respectively [67]. The presence of these bands (VI, VII) suggests the ionic character of all samples [57]. The relative areas of these bands are bigger for the glass system with BaO than with CaO or SrO. Therefore, an increase in the quantity of non-bridging oxygen ions due to depolymerization of the glass network can be observed primarily for BaO as oxide modifier. Furthermore, the intensity of band (VIII) at about 1161–1182 cm^−1^ attributed to the asymmetric stretching of PO_2_^−^ in the Q^2^ structure [67] significantly decreases when oxide modifiers MO change in direction CaO < SrO < BaO. The obtained results indicate that when glass modifiers are changed in glass host the conversion of Q^2^ to Q^1^ units occurs. The IR band near 1250 cm^−1^ appears due to overlapping of the asymmetric stretching vibration of P–O bond with the asymmetric stretching vibration of PO_2_^−^ mode in Q^2^ units. This band (IX) may be evidence of the stretching mode of P=O double bonds in the phosphate structural units [68]. On the contrary, the band (X) at about 1300 cm^−1^ is associated with the harmonic of the doubly bonded oxygen vibration (P=O) [58]. Table 1 presents the infrared bands observed for studied phosphate glasses.

**Figure 4:**
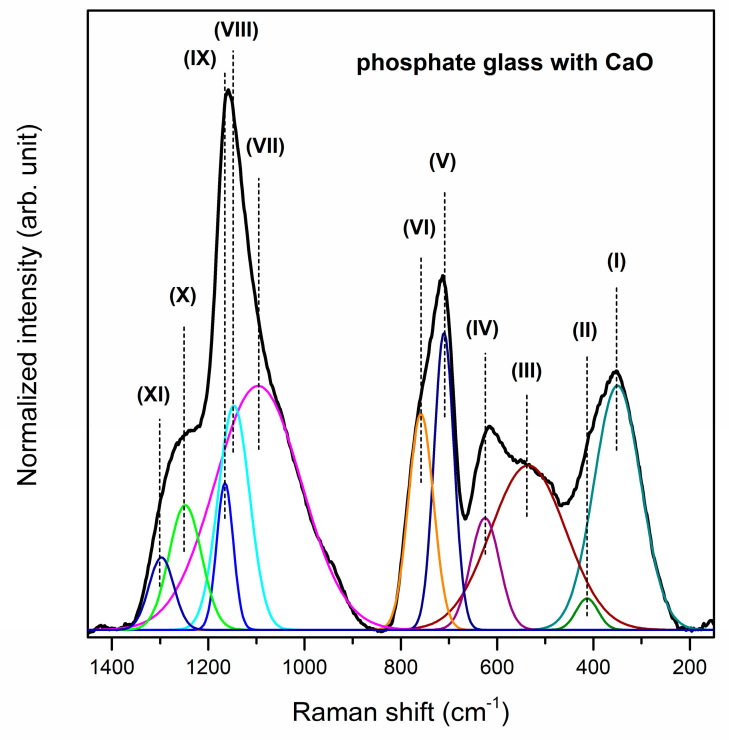
Deconvoluted Raman spectra of phosphate glass with CaO as oxide glass modifier.

**Figure 5:**
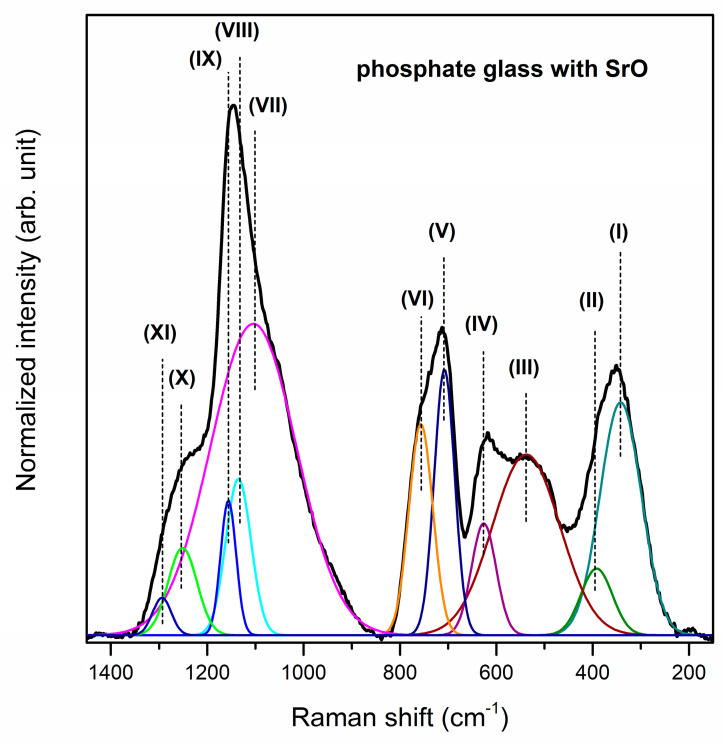
Deconvoluted Raman spectra of phosphate glass with SrO as oxide glass modifier.

**Figure 6:**
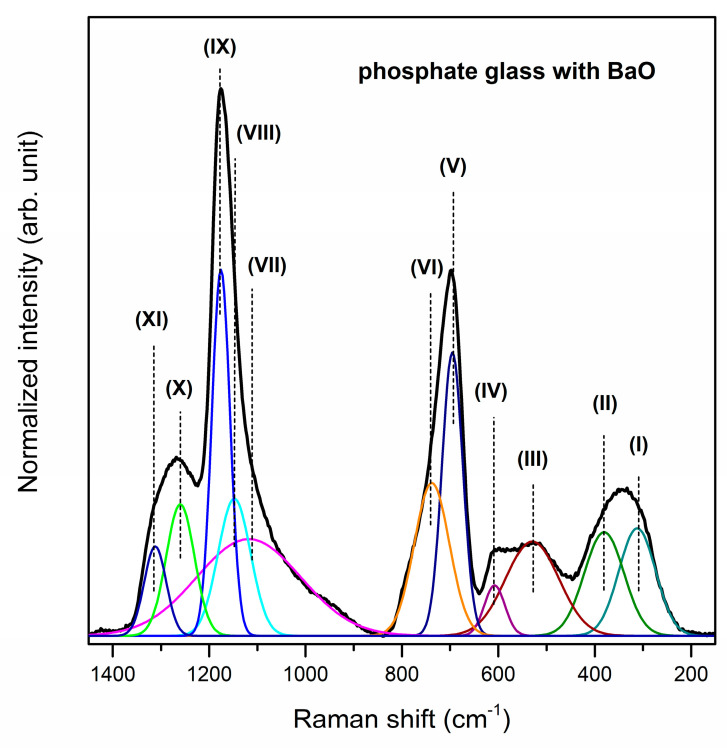
Deconvoluted Raman spectra of phosphate glass with BaO as oxide glass modifier.

The Raman spectra of studied phosphate systems with glass modifiers were registered in the 150–1450 cm^−1^ spectral region. Also for Raman spectra, it was necessary to deconvolute the spectra for better understanding the influence of calcium oxide, strontium oxide and barium oxide addition on the glass structure (Figure 4, Figure 5 and Figure 6). The observed bands were assigned based on literature data [55,58,61,68,69,70,71,72]. Generally, all spectra can be divided into two main broad and unresolved vibration band groups located in the 200–800 cm^−1^ and 850–1400 cm^−1^ frequency regions. Furthermore, bands above 1300 cm^−1^ corresponding to the Q^3^ units are not registered for analyzed glass systems. Therefore, it can be concluded that there are no Q^3^ units in the structure of the studied phosphate glasses [55]. The bands near at ~350 cm^−1^ and ~400 cm^−1^ confirmed the presence of gallium oxide in the obtained glass composition because systems containing tetrahedrally coordinated Ga atoms give intense low-frequency Raman bands assignable to bending modes of mixed Ga–O–M bridges [61]. The first one (band (I)) can be attributed to the GaO_6_ vibrational groups [61]. 

The second one (band (II)) probably correspond to symmetric bending vibrations of Ga–O–P linkages [61,69] or bending vibrations of PO_4_ units [68]. The band (III) at about 520 cm^−1^ corresponds to bending vibrations of P_2_O_7_^4−^ groups [58]. Moreover, the band (IV) centered at 625/626/607 cm^−1^ is assigned to the symmetric stretching vibrations of P–O– terminal bonds [70]. It is important to note that the intensity of the band at about 400 cm^−1^ increases, whereas the intensity of bands located at 350, 520, 625 cm^−1^ decreases when oxide modifiers MO change in direction CaO < SrO < BaO. The intense Raman bands at around 710 cm^−1^ (V) and 758 cm^−1^ (VI) are attributed to the symmetric stretching vibrations of P–O–P bonds in Q^2^ metaphosphate units and the symmetric stretching vibrations of P–O–P associated with Q^1^ tetrahedral units, respectively [70]. Additionally, the band at 710 cm^−1^ is more intense than the band near at 758 cm^−1^. Therefore, it can be stated that in the structures of the studied phosphate systems with oxide modifiers CaO, SrO and BaO, phosphorus tetrahedra are linked into chains and rings, and presumably Q^2^ units prevail [71]. 

Based on the analysis of the Raman spectra in the 850–1400 cm^−1^ frequency regions it can be assumed that conversion structural units occurs (Q^1^ to Q^2^). The band (VII) located at around 1096/1104/1117 cm^−1^ determines the formation of Q^1^ phosphate tetrahedra [55]. On the other hand, the bands (VIII, IX) corresponding to the symmetric stretching mode of O–P–O non-bridging oxygens, indicating the formation of metaphosphate structural units (Q^2^), were registered in the 1135–1176 cm^−1^ frequency regions [70,72]. The relative areas and intensities of these bands (VII–IX) significantly depend on the type of oxide glass modifiers MO (M = Ca, Sr, Ba). For the glass system with BaO, the intensity and area of band attributed to symmetric stretching vibrations of P–O bonds in pyrophosphate units (Q^1^) are lower than other obtained glasses. However, the most intense bands due to the formation of Q^2^ phosphate tetrahedral are observed for phosphate glass with BaO as oxide modifier. For that reason, it could be concluded that the conversion of pyrophosphate tetrahedra (Q^1^) to metaphosphate (Q^2^) tetrahedras occurs when oxide modifiers MO change in direction CaO < SrO < BaO. The last two weak Raman bands may correspond to the symmetric stretching of P–O bonds [69] or asymmetric stretching vibrations of PO_2_^−^ groups in Q^2^ units [70,71] Its assignment due to the symmetric stretch of P=O terminal oxygens is also taken into consideration [71]. Table 2 presents all registered bands in the Raman spectra for studied phosphate glasses.

### 3.2. Spectroscopic Properties of Phosphate Glasses

**Figure 7:**
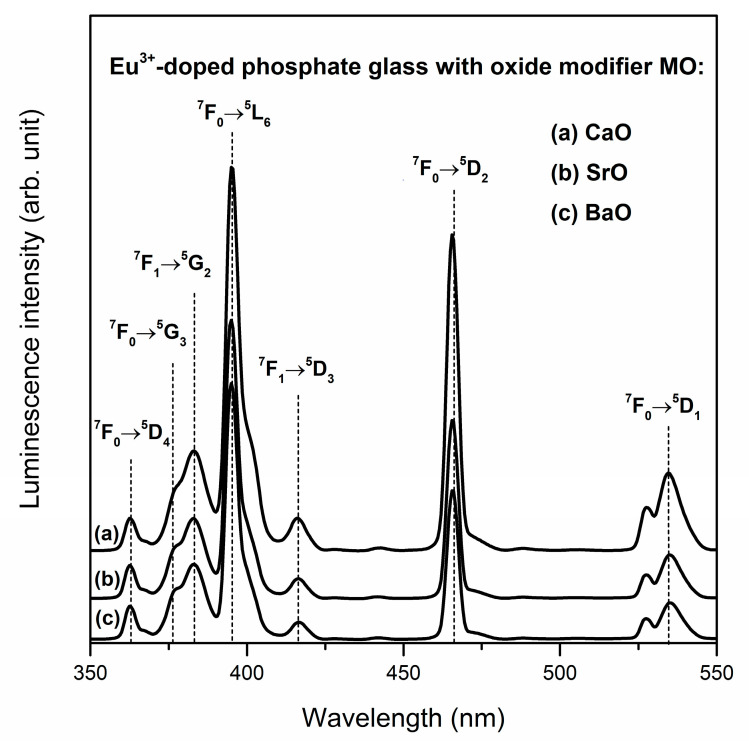
Excitation spectra for Eu^3+^-doped phosphate glasses with oxide glass modifiers MO.

Figure 7 shows the excitation spectra of Eu^3+^-doped phosphate systems with oxide modifiers MO. These spectra were monitored at λ_em_ = 611 nm. 

The recorded excitation bands originating with the transition from the ^7^F_0_ and ^7^F_1_ states to higher-lying states are narrow and well resolved. Independently on the kind of MO, the among registered bands the most intense bands correspond to ^7^F_0_→^5^L_6_ (395 nm) and ^7^F_0_→^5^D_2_ (465 nm) transitions. Therefore, the Eu^3+^ ions that present in the glass host could be excited from the ^7^F_0_ ground state through UV or visible radiation into one of the higher-lying levels.

**Figure 8:**
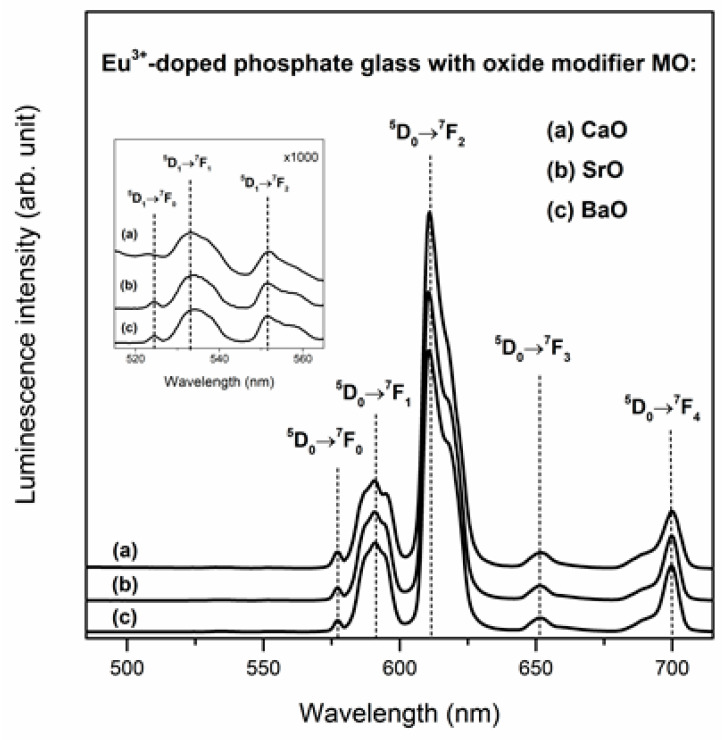
Luminescence spectra for Eu^3+^-doped phosphate glasses with oxide glass modifiers MO.

Figure 8 presents the emission spectra of Eu^3+^ ions in investigated phosphate glasses. For all samples, spectra were registered under excitation λ_exc_ = 395 nm. Because of the small energy gaps between ^5^L_6_, ^5^D_3_, ^5^D_2_, ^5^D_1_ states, the excitation energy was transferred non-radiatively to the ^5^D_0_ level. On the other hand, the energy gaps between the ^5^D_0_ and ^7^F_J_ (where J = 0, 1, 2, 3, 4) levels are quite large. Consequently, the visible luminescence due to ^5^D_0_→^7^F_J_ transitions of Eu^3+^ ions are possible. The intense emission bands located at 577, 591, 611, 651 and 700 nm are assigned to the ^5^D_0_→^7^F_0_, ^5^D_0_→^7^F_1_, ^5^D_0_→^7^F_2_, ^5^D_0_→^7^F_3_ and ^5^D_0_→^7^F_4_ transitions, respectively. Moreover, there are additional weak bands in the range 520-560 nm (inset for Figure 8). These visible luminescence bands correspond to the ^5^D_1_→^7^F_0_, ^5^D_1_→^7^F_1_ and ^5^D_1_→^7^F_2_ transitions of Eu^3+^ ions. The emission spectra were normalized to the ^5^D_0_→^7^F_1_ transitions (590 nm) in order to better comparison. It was stated that the intensity of band due to ^5^D_0_→^7^F_2_ depends significantly on the oxide modifiers. It is generally known that the ^5^D_0_→^7^F_1_ is a magnetic dipole (MD) transition which is independent of local symmetry [73]. However, ^5^D_0_→^7^F_2_ is strongly influenced by the environment of europium ions and this electric dipole (ED) transition is said to be a hypersensitive transition [74]. For that reason, the ratio of the ^5^D_0_→^7^F_2_ to ^5^D_0_→^7^F_1_ transitions informs us about local asymmetry around the Eu^3+^ ions in the glass host. The ratio of integrated emission intensity of these transitions is defined as the red-to-orange fluorescence intensity ratio R/O [75]. It was found that the value of R/O depends on the modifiers (calcium oxide, strontium oxide and barium oxide) in studied phosphate glass systems. The value of the fluorescence intensity ratio decreases in direction CaO < SrO < BaO. The value of R/O factor is the smallest for glass with BaO (3.09), whereas for the system with CaO (3.77) is the highest. For that reason, the local asymmetry around the europium ions and the covalent character of Eu–O bond in phosphate glasses increase in the same direction. 

**Figure 9:**
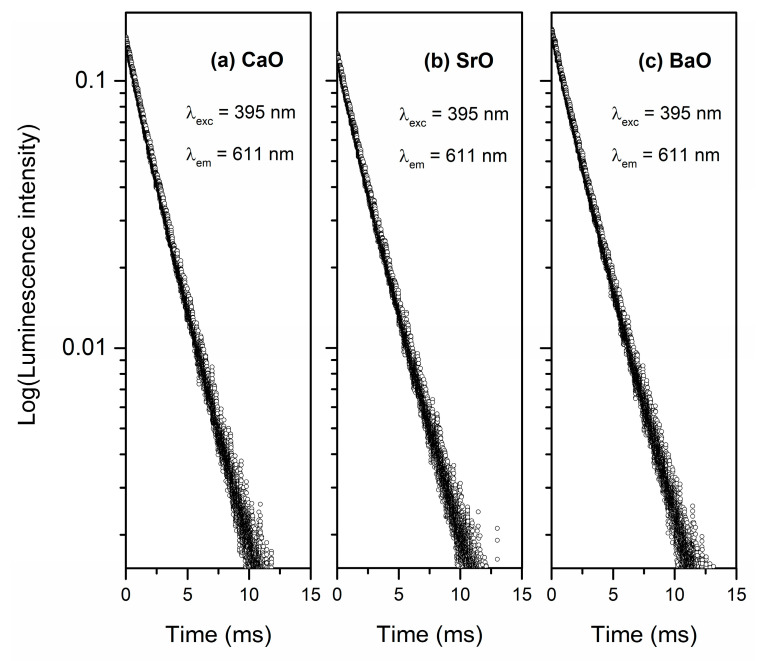
Luminescence decay curves for ^5^D_0_ state of Eu^3+^ ions in phosphate glass with oxide glass modifiers MO.

Next, luminescence decays from the upper ^5^D_0_ excited level of europium ions in the studied systems were performed. Figure 9 shows the decay curves for phosphate glasses with modifiers CaO, SrO and BaO. Based on decays, the luminescence lifetime for ^5^D_0_ state of Eu^3+^ was calculated. It is noteworthy that the obtained results for phosphate glasses indicate the influence of modifier MO on the value of lifetime. However, its value is reduced from 2.20 ± 0.0013 ms for glass with BaO to 2.06 ± 0.0013 ms for the sample with CaO. In contrast to the fluorescence intensity ratio R/O, the ^5^D_0_ lifetime of europium ions increases in direction CaO < SrO < BaO. The similar trend was obtained for europium ions in tellurophosphate glasses and lithium borate glasses [76,77]. According to Jha et al. [66] the highest lifetime value of the ^5^D_0_ state of Eu^3+^ for glass with BaO as oxide modifier may suggest a lesser cross-relaxation effect between rare earth ions due to the greater depolymerization of phosphate network, resulting from an increase in the non-bridging oxygen (NBO) group. This effect is in good agreement with our analysis of near-infrared spectra (Figure 1, Figure 2 and Figure 3) which confirm that the poly-phosphate structure dominated by metaphosphate (Q^2^) and pyrophosphate (Q^1^) units and maximum depolymerization was observed for glass with barium oxide network modifier. Moreover, it was stated that the value of luminescence lifetime for phosphate glass systems doped with Eu^3+^ ions increases with an increasing ionic radius and atomic weight of oxide modifiers (CaO < SrO < BaO). On the contrary, Hermann et al. [78] investigated silicate systems with different network modifier oxides MO (CaO, SrO, BaO) and shown that ^5^D_0_ luminescence lifetime of europium ions increases with an increasing atomic weight of MO.

**Figure 10:**
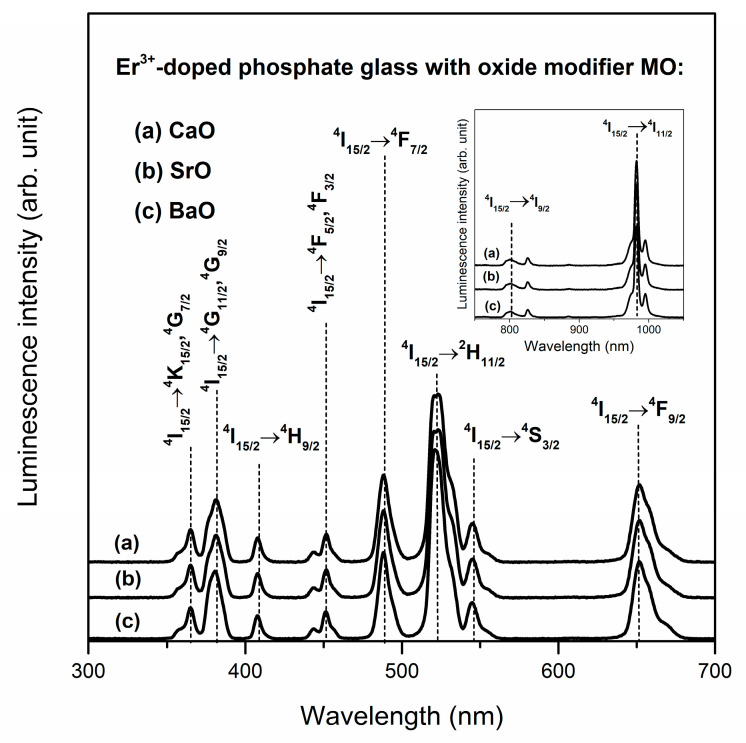
Excitation spectra for Er^3+^-doped phosphate glasses with oxide glass modifiers MO.

Phosphate glasses doped with erbium ions were investigated in order to analyze influence of oxide glass modifiers MO (M = Ca, Sr, Ba) on the spectroscopic properties of systems for near-infrared photonic applications. The excitation spectra were monitored at λ_em_ = 1535 nm. This wavelength is related to the most typical near-infrared emission for Er^3+^ ions. The observed bands originating to transitions from ^4^I_15/2_ ground state to the higher-lying excited states of erbium ions are shown in Figure 10. Based on the recorded spectra it was found that independently on the kind of oxide modifier used, the erbium ions could be excited through UV, visible or near-infrared radiation. In the wavelength range of 300–700 nm the intense bands due to ^4^I_15/2_→^4^F_7/2_ (488 nm), ^4^I_15/2_→^2^H_11/2_ (523 nm), ^4^I_15/2_→^4^F_9/2_ (652 nm) transitions of Er^3+^ ions were registered. 

**Figure 11:**
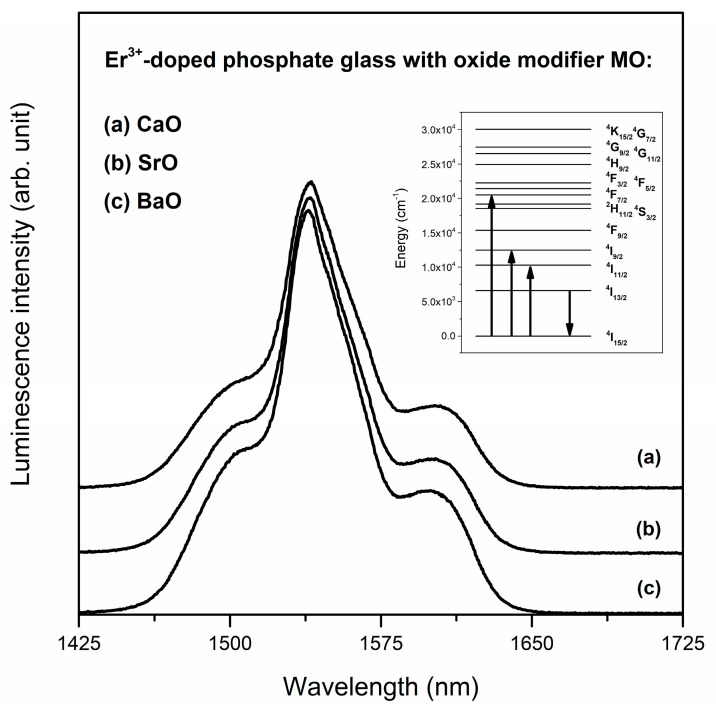
Luminescence spectra for Er^3+^-doped phosphate glasses with oxide glass modifiers MO.

Additionally, weak bands corresponding to excitation erbium ions from ^4^I_15/2_ ground state to the higher-lying states (^4^K_15/2_, ^4^G_7/2_, ^4^G_11/2_, ^4^G_9/2_, ^4^H_9/2_, ^4^F_5/2_, ^4^F_3/2_, ^4^S_13/2_) were observed. It is worth noting that recording of excitation bands in the near-infrared spectral range (800 and 980 nm) indicates that studied glass systems doped with Er^3+^ ions could be also directly pumped by commercially available laser diodes (LD; inset of Figure 10). Taking this into consideration, the emission spectra were registered under direct excitation by 488 nm (^4^F_7/2_ state) and 980 nm (^4^I_11/2_ state). Regardless of the excitation wavelength, the near-infrared emission spectra consist of a broad luminescence band at about 1.5 μm corresponding to ^4^I_13/2_→^4^I_15/2_ laser transition of Er^3+^ ions (Figure 11). It is important to note that potential applications of glass materials for optoelectronic devices require an in-depth analysis of two important parameters. The first is the luminescence linewidth for ^4^I_13/2_→^4^I_15/2_ transition of Er^3+^ ions. This spectroscopic parameter is described as the full width at half maximum (FWHM) and it is practically independent of the presence of calcium oxide, strontium oxide or barium oxide in phosphate glass host. The differences between values of FWHM for samples containing metal oxides are comparably small (Table 3.). Moreover, it was stated that the luminescence linewidth values obtained for near-infrared laser transition of erbium ions in studied phosphate glasses are similar to other glass systems based on P_2_O_5_ [79,80].

**Figure 12:**
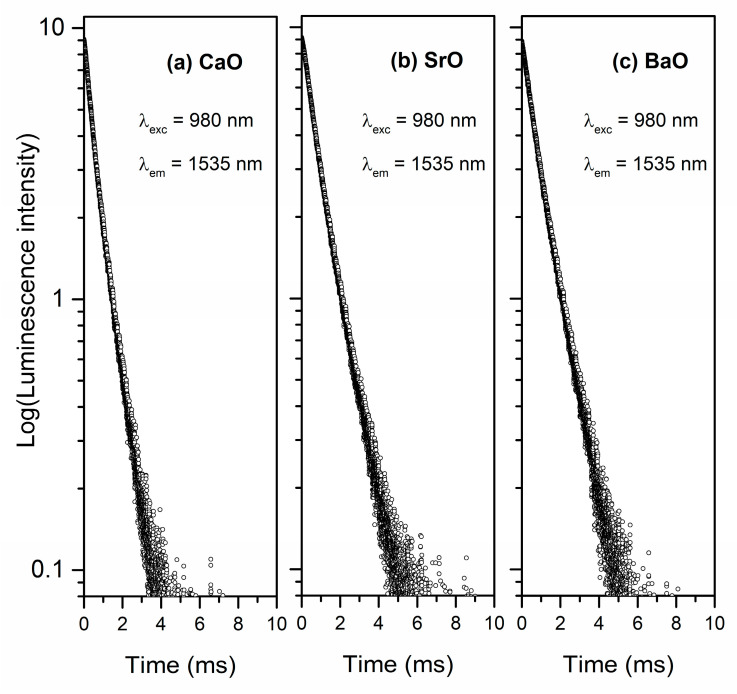
Luminescence decay curves for ^4^I_13/2_ state of Er^3+^ ions in phosphate glasses with oxide glass modifiers MO.

Lastly, luminescence decay curves for ^4^I_13/2_ state of erbium ions in studied systems were recorded at λ_exc_ = 980 nm and λ_em_ = 1535 nm and based on them, the luminescence lifetimes were found out. Independently of glass modifiers (CaO, SrO or BaO), registered decay profiles are nearly exponential (Figure 12). The value of the luminescence lifetime for ^4^I_13/2_ state of Er^3+^ ions is higher for glass with BaO (τ_m_ = 920 ± 0.71 μs) than for sample with SrO. However, the phosphate system with CaO as glass oxide modifiers is characterized by the lowest luminescence lifetime (τ_m_ = 640 ± 0.66 μs). Therefore, the value of luminescence lifetime for ^4^I_13/2_ state of Er^3+^ ions in studied phosphate systems increased in the direction CaO < SrO < BaO. It is interesting to see that the value of luminescence lifetime for phosphate glass systems doped with Er^3+^ ions increases with an increasing ionic radius of oxide modifiers MO. On the other hand, based on the results obtained for germanate glasses Wang et al. [81] showed that the radiative lifetime of erbium ions at the ^4^I_13/2_ level is decreased, when the radius of alkaline earth ion increases. 

Considering the above results that obtained phosphate systems with CaO, SrO and BaO are promising materials for visible and near-infrared photonic applications. Table 4 and Table 5 present a comparison of spectroscopic parameters in various glass hosts doped with europium and erbium ions, respectively. 

If we compare the spectroscopic properties determined for systems doped with Eu^3+^ ions, it can be stated that due to the highest value of luminescence lifetime for ^5^D_0_ state, the glass with BaO is a good candidate for red-emitting materials, that may find potential use in photonic devices. According to previous studies, our phosphate system is characterized by longer luminesce lifetime than lead-free borate and germanate glasses [82,83]. The value of lifetime for ^5^D_0_ state of Eu^3+^ ions in phosphate glass is close to the value evaluated for borate (1.81 ms) and silicate (2.17) systems with BaO as oxide modifier [77,78].

Moreover, the luminescence lifetime for glass with BaO is significantly longer than the value of the lifetime for the ^5^D_0_ state for heavy metal oxide systems doped with Eu^3+^ ions [86,87,88,89]. Also, the results obtained for glasses containing with Er^3+^ ions indicate that among our studied samples the glasses with BaO are the best materials, that may be applied as near-infrared broadband amplifiers. It is well known that there are two important factors in the success of glasses as optical amplifiers. The first is the high value of full width in half maximum (FWHM) while the second is relatively long luminescence lifetime of erbium ions. In comparison to other oxide phosphate glasses [79,80] the FWHM evaluated for the studied system is wider (44 nm). On the other hand, the value of this spectroscopic parameters is significantly lower than those of borate [83] and silicate glasses [90]. In case of luminescence decays from ^4^I_13/2_ state of Er^3+^ ions it can be concluded that the lifetime value in studied phosphate glass is comparable to results obtained for phosphate [80] and silicate systems [90]. However, numerous studies have shown that the values of lifetimes are dependent critically on the glass composition and it is possible to synthesize the more efficiently-emitted materials [79,83,91].

## 4. Conclusions

In summary, the influence of oxide modifiers on multiple properties (structural and spectroscopic) of phosphate systems singly doped with rare earth ions (Eu^3+^ and Er^3+^) was studied. Based on near-infrared (IR-ATR) and Raman spectroscopy it has been proven that a change of modifier (CaO, SrO and BaO) in the glass host cause an increase in the number of Q^1^ and Q^2^ tetrahedra with non-bridging oxygens due to the depolymerization of the phosphate network. Independently on the kind of oxide modifier used intense red (611 nm) and near-infrared emissions (1.5 μm) were observed for glasses doped with Eu^3+^ and Er^3+^ ions, respectively. It was demonstrated that glass modifiers greatly effect on the spectroscopic parameters of rare earth ions in the studied systems. Firstly, the value of fluorescence intensity ratio R/O for europium ions indicates a high degree of covalence between Eu^3+^ and O^2–^, and the local asymmetry around the europium ions in the glass host. Moreover, the value of R/O parameter decreases, whereas the ^5^D_0_ lifetime of europium ions increases in direction CaO < SrO < BaO. The effect of glass modifiers on luminescence linewidth for 1.5 μm emission (FWHM) was not clearly observed. The luminescence lifetime for ^4^I_13/2_ state of erbium ions change in towards with increasing of the radius of alkaline earth ion CaO < SrO < BaO. Our results suggest the applicability of the phosphate glasses with oxide modifiers as potential red and near-infrared photoluminescent materials in photonic devices. Especially, the systems with BaO show good luminescence properties and we can conclude that these rare earth-doped phosphate glasses are promising materials for visible and near-infrared photonic applications.

## Figures and Tables

**Table 1:** Band positions and their assignments.

Band	Frequency (cm^−1^)	Band Assignment	References
(I)	499/488/452 cm^−1^	harmonics of bending vibrations of O=P–O linkages	[57]
(II)	640/639/588 cm^−1^	stretching vibrations the M–O–P bonds stretching vibrations of P–O–P mode bending vibrations of O–P–O modes	[58,59]
(III)	708/725/722 cm^−1^	symmetric stretching vibrations of P–O–P linkages in between Q^1^ and Q^2^	[58]
(IV)	772/772/784 cm^−1^	symmetric stretching mode of P–O–P bonds	[62]
(V)	910/891/877 cm^−1^	asymmetric stretching vibrations of bridging oxygen atoms in P–O–P bonds asymmetric stretching vibrations of the P–O–P linkage of Q^1^ and Q^2^ tetrahedra with non-bridging oxygen	[63,64]
(VI)	1006/1009/949 cm^−1^	asymmetric stretching vibrations of PO_4_^3−^ structural group	[66]
(VII)	1086/1087/1079 cm^−1^	symmetric stretching vibrations of PO_4_^3−^ tetrahedral (PO^−^ ionic group) symmetric stretching vibrations of PO_3_^2−^ in the Q^1^ tetrahedra	[66,67]
(VIII)	1173/1182/1161 cm^−1^	asymmetric stretching vibrations of PO_2_^−^ in the Q^2^ tetrahedra	[67]
(IX)	1255/1258/1240 cm^−1^	P=O stretching vibration of PO_2_^−^ groups	[68]
(X)	1304/1301/1303 cm^−1^	harmonic of the doubly bonded oxygen vibration	[58]

**Table 2:** Band positions and their assignments.

Band	Frequency (cm^−1^)	Band Assignment	References
(I)	350/342/312 cm^−1^	GaO_6_ vibrational groups	[69]
(II)	413/392/381 cm^−1^	Ga-O-P linkages bending vibrations of PO_4_ units	[68,69]
(III)	535/538/530 cm^−1^	bending vibrations of P_2_O_7_^4−^ groups	[58]
(IV)	625/626/607 cm^−1^	symmetric stretching vibrations of P–O– terminal bonds	[70]
(V)	710/708/695 cm^−1^	symmetric stretching vibrations of P–O–P bonds in Q^2^ metaphosphate tetrahedra	[70]
(VI)	758/757/738 cm^−1^	symmetric stretching vibrations of P–O–P bonds associated with Q^1^ tetrahedra	[55,70]
(VII)	1096/1104/1117 cm^−1^		
(VIII)	1147/1135/1148 cm^−1^	symmetric stretching modes of non-bridging atoms on Q^2^ tetrahedra	[72]
(IX)	1165/1156/1176 cm^−1^		
(X)	1248/1251/1260 cm^−1^	symmetric stretching of P–O bonds	[69]
(XI)	1297/1284/1312 cm^−1^	stretching vibrations of non-bridging bonds PO_2_^−^ of Q^2^ metaphosphate tetrahedra P=O stretching of terminal oxygen	[71]

**Table 3:** Spectroscopic parameters for Ln^+^-doped phosphate glasses.

Ln^3+^	Spectroscopic Parameter	Oxide Glass Modifier		
		CaO	SrO	BaO
Eu^3+^	λ_max_^5^D_0_→^7^F_1_ (nm)	590.5	590.5	591.0
	λ_max_^5^D_0_→^7^F_2_ (nm)	611.0	610.5	611.0
	R/O	3.77	3.29	3.09
	τ_m_ (ms)	2.06 ± 0.0013	2.15 ± 0.0015	2.20 ± 0.0013
Er^3+^	λ_max_^4^I_13/2_→^4^I_15/2_ (nm)	1540	1540	1539
	FWHM (nm)	44	43	44
	τ_m_ (μs)	640 ± 0.66	888 ± 0.65	920 ± 0.71

**Table 4:** Comparison of luminescence lifetime for ^5^D_0_ state (Eu^3+^) of inorganic glasses.

Glass Composition	τ_m_ for ^5^D_0_ State of Eu^3+^ (ms)	References
P_2_O_5_-Ga_2_O_3_-BaO-Eu_2_O_3_	2.20 ± 0.0013	present work
B_2_O_3_-Ga_2_O_3_-BaO-Eu_2_O_3_	1.60	[82]
GeO_2_-Ga_2_O_3_-BaO-Eu_2_O_3_	1.22	[83]
Li_2_O-BaO-B_2_O_3_-Eu_2_O_3_	1.81	[77]
SiO_2_-Al_2_O_3_-BaO-Eu_2_O_3_	2.17	[78]
SiO_2_-MgO-CaO-Na_2_O-K_2_O-Eu_2_O_3_	2.55	[84]
TeO_2_-La_2_O_3_-TiO_2_-Eu_2_O_3_	0.82	[85]
PbO-P_2_O_5_-Ga_2_O_3_-Eu_2_O_3_	2.02	[86]
PbO-SiO_2_-Ga_2_O_3_-Eu_2_O_3_	1.27	[87]
La_2_O_3_-Bi_2_O_3_-B_2_O_3_-Eu_2_O_3_	1.01	[88]
La_2_O_3_-PbO-B_2_O_3_-Eu_2_O_3_	1.29	[88]
Bi_2_O_3_-GeO_2_-Eu_2_O_3_	1.03	[89]

**Table 5:** Comparison of parameters FWHM for band at 1.5 μm (Er^3+^), luminescence lifetime for ^4^I_13/2_ state (Er^3+^) of inorganic glasses.

Glass Composition	FWHM (nm)	τ_m_ for ^4^I_13/2_ State of Er^3+^ (ms)	References
P_2_O_5_-Ga_2_O_3_-BaO-Er_2_O_3_	44	0.92 ± 0.0071	present work
B_2_O_3_-Ga_2_O_3_-BaO-Er_2_O_3_	98	0.42	[82]
GeO_2_-Ga_2_O_3_-BaO-Er_2_O_3_	50	5.35	[83]
P_2_O_5_-Li_2_O-Al_2_O_3_-BaO-MgO-Gd_2_O_3_-Er_2_O_3_	30	7.01	[79]
P_2_O_5_-K_2_O-BaO-Al_2_O_3_-Yb_2_O_3_-Er_2_O_3_	37	0.78	[80]
SiO_2_-GeO_2_-CaO-BaO-Nb_2_O-Li_2_O-Er_2_O_3_	77	0.78	[90]
TeO_2_-ZnO-BaO-Er_2_O_3_	46	4.70	[91]
PbO-P_2_O_5_-Ga_2_O_3_-Er_2_O_3_	52	2.50	[92]
